# Influence of differential source patterns in the detection of signals of disproportionate reporting for PARP inhibitors

**DOI:** 10.3389/fdsfr.2024.1497116

**Published:** 2024-12-06

**Authors:** Jordi Mestres

**Affiliations:** ^1^ Institut de Quimica Computacional i Catalisi (IQCC), Facultat de Ciencies, Universitat de Girona, Girona, Spain; ^2^ Chemotargets SL, Parc Científic de Barcelona, Barcelona, Spain

**Keywords:** disproportionality analyses, signal detection, PARP drugs, reporting patterns, pharmacovigilance

## Abstract

**Introduction:**

Current individual case safety report (ICSR) databases contain almost 56 million unique spontaneous declarations of drug-event associations by health professionals but also by patients themselves. These databases have become a useful source for detecting signals of disproportionate reporting (SDR). However, since health professionals use a medical jargon that is often distant from the more colloquial terms used by patients, they usually report more frequently certain adverse events than patients and *vice versa*. The main objective of this work is to illustrate the existence of different reporting patterns among drugs within a class and to analyze their potential impact on SDR detection.

**Methods:**

Four ICSR databases were considered, namely, FAERS, VAERS, JADER, and VigiBase, with reports up until March 2024. They were all integrated in a single database following a careful deduplication and COVID-19 correction protocol. Measures of reporting odds ratio, proportional reporting ratio and empirical Bayesian geometric mean were used to evaluate disproportionate reporting.

**Results:**

The reporting patterns of four marketed oncology drugs, namely, olaparib, rucaparib, niraparib, and talazoparib, and an investigational drug, veliparib, were compared to those of a diverse set of eight clinically observed SDR, namely, fatigue, asthenia, anaemia, thrombocytopenia, neutropenia, insomnia, intestinal obstruction, and pneumonitis. The source pattern analysis revealed that olaparib and talazoparib are most frequently reported by physicians, and physicians are the main reporters of events such as neutropenia and pneumonitis, predisposing these events to be detected as SDR for those PARP inhibitors. In contrast, rucaparib and niraparib are most frequently reported by American consumers, and American consumers are the main reporters of events such as insomnia and intestinal obstruction, facilitating their detection as SDR for those two drugs. SDR detection was found to be robust to ICSR data completeness.

**Discussion:**

Matched reporting patterns between drugs and events may predispose certain drugs to be disproportionally associated with adverse events. Therefore, SDR detected from matched drug-event source patterns in ICSR databases should be challenged during signal validation. Class SDR for drugs with differential source patterns (such as fatigue, asthenia, anaemia, thrombocytopenia, and neutropenia for all PARP inhibitors) usually involve correcting opposite drug-event source patterns.

## 1 Introduction

Spontaneous reporting systems (SRS) have become popular sources for signal detection in pharmacovigilance (Raschi et al., 2018). Compared to the limited number and geographic locality of patients involved in clinical trials, the main strength of SRS is that they collect a large volume of individual case safety reports (ICSR) from all over the world deposited spontaneously not only by health professionals (physicians, pharmacists) but also by patients themselves (consumers) and even lawyers (Rodriguez et al., 2001). By evaluating the reporting frequency of a specific adverse event associated with a particular drug, in the context of all other drugs, one can extract signals of disproportionate reporting (SDR), that is, adverse events that for a particular drug have a probability of being reported more often than any other adverse event compared to the probability for reference drugs (Deshpande et al., 2010).

However, disproportionality analyses on ICSR databases are subject to several biases that, if not identified and addressed properly, limit the validity of the approach for signal detection (Michel et al., 2017; Cutroneo et al., 2024; Fusaroli et al., 2024). Among them, reporting biases are of common concern, including both over-reporting, by novelty (Weber, 1984), competition (Arnaud et al., 2016), notoriety (Pariente et al., 2007), seriousness (Moulis et al., 2012) or masking (Montes-Grajales et al., 2023), and under-reporting (Hazell and Shakir, 2006).

Beyond frequency, reporting biases can be also analyzed from the perspective of the original source of the report, both the reporter type and the world region. For example, it has been shown that consumers are more likely than health professionals to report adverse events linked to psychiatric disorders and less likely to report events from the blood and lymphatic system (Aagaard et al., 2009; Al Dweik et al., 2020).

Accordingly, the main objective of this work is to investigate the presence of source biases among drugs of a given class and the influence of differential reporting patterns on the SDR detected. The drug class of poly-ADP ribose polymerases (PARP) inhibitors (Makin, 2021) is taken as an illustrative use case.

## 2 Materials and methods

### 2.1 Spontaneous reporting systems

Four post-marketing databases of spontaneous reports (updated to 31 March 2024) were used in this study, namely, the United States Food and Drug Administation (FDA) Adverse Event Reporting System (FAERS, 2024) and Vaccine Adverse Event Reporting System (VAERS, 2024), the Japan Adverse Drug Event Report (JADER) collected by the Pharmaceuticals and Medical Devices Agency (PMDA, 2024) and the World Health Organisation (WHO) Global Database of Individual Case Safety Reports, VigiBase (Lindquist, 2008), managed at the Uppsala Monitoring Center (UMC), that contain a total of 23,880,384, 2,608,250, 894,123 and 38,259,277 reports, respectively. Prior to performing any report extraction and statistical analysis, all duplicate reports within and between databases were removed (*vide infra*), which resulted in a final number of 55,751,007 unique reports distributed as 17,651,271, 2,238,340, 851,215 and 35,010,181 unique reports from FAERS, VAERS, JADER and VigiBase, respectively. All analyses were performed with ClarityVista (ClarityVista 2024).

### 2.2 Data extraction and treatment

All FAERS and VAERS reports were downloaded quarterly from the FDA websites (FAERS Quarterly Data Extract Files 2024), whereas JADER reports were obtained from INTAGE Healthcare (INTAGE Healthcare, 2024) and VigiBase reports from WHO-UMC (WHO-UMC, 2024). VigiGrade scores (Bergvall et al., 2014) were calculated for all reports in all sources and median vigiGrade values were used to reflect the overall content completeness of ICSR for drugs and events.

Raw names of drug products included in reports were extracted from the drug_name and prod_ai fields of the drug table in FAERS, from the and vax_name field of the vax table in VAERS, from the drugname_eng field of the drug table in JADER, and from the substance_name and drug_name fields of the mp and sun tables in VigiBase. All drug name terms extracted from those fields were then first processed to detect the presence of strings of drug names in our drug thesaurus and then manually inspected and mapped to a drug main name. The focussed list of marketed drugs considered in this study included olaparib (lynparza), rucaparib (rubraca), niraparib (zejula), talazoparib (talzenna), to which the investigational drug veliparib (ABT-888) was added for completeness. For adverse events, preferred terms defined in the Medical Dictionary for Regulatory Activities (MedDRA^®^ version 23.0) were used.

Raw names for adverse events included in reports were obtained from the pt (preferred term) field of the reaction table in FAERS, from the symptom (N = 1–5) fields of the symptoms table in VAERS, from the adr_code field of the reaction table in JADER, and from the meddra_id field of the adr table in VigiBase. Then, all event terms were mapped to preferred terms in MedDRA and assigned to its corresponding system organ class (SOC).

Duplicate detection involves comparing the contents of the following fields: sex, age, region, lists of drugs, indications, and events, start and event dates, time-to-onset values, and suspiciousness assignments. Accordingly, FAERS and VAERS reports having the same contents in those fields were removed as duplicates. Also, for these two sources, reports from the same case with a time difference below the calculated time interval for subsequent reports of the same case and with coincident information in the rest of the fields were considered replicates and assigned as duplicates. VigiBase facilitates the removal of duplicate reports in FAERS and VAERS by providing record identifiers in each database. Finally, duplicates between JADER and the other sources were identified by comparing the contents of those fields containing corresponding data. If duplicates and/or replicates of reports coming from one or multiple sources are identified, only the report from the source with the earliest report date is retained for the statistical analyses. All ICSR databases were also corrected to mitigate the masking effects from COVID-19 vaccine reports (Montes-Grajales et al., 2023).

At the end of these processes, the total combined number of unique ICSR extracted for olaparib, rucaparib, niraparib, talazoparib and veliparib was 22,432, 8,806, 26,954, 2,090 and 791, respectively.

### 2.3 Drug indications and approval dates

First drug approval dates and indications are collected in Table 1. Olaparib was the first PARP inhibitor to be approved in 2014 for ovarian cancer by both the FDA and the European Medicines Agency (EMA) and in 2019 by the Japan Pharmaceuticals and Medical Devices Agency (PMDA). It has since received approval by these three agencies for extended use in other cancer types, including breast cancer (2018), pancreatic cancer (2019) and prostate cancer (2020). In 2016, rucaparib was approved by the FDA for ovarian cancer and later granted extended use in prostate cancer (2020). Shortly after (2017), niraparib also received approval for ovarian cancer. Finally, talazoparib was approved for treatment of breast cancer in 2018 and recently received approval for extended use in prostate cancer (2023).

**TABLE 1:** First approval dates by the different regulatory agencies of PARP drugs for various organ cancers (source: https://hemonc.org/). For every drug and indication, also added are the total number of individual case safety reports (ICSR) and the relative percentages of female and male patients (in parenthesis).

Indication	Drug	ICSR (%Female/%Male)	Agency	Approval date
Ovarian cancer	Olaparib Rucaparib Niraparib	10,204 (97%/0%) 5,189 (96%/0%) 16,675 (65%/0%)	FDA EMA PMDA FDA EMA FDA EMA PMDA	2014-12-19 2014-12-16 2019-06-18 2016-12-19 2018-05-23 2017-03-27 2017-11-16 2020-09-29
Breast cancer	Olaparib Talazoparib	2,068 (91%/2%) 1,013 (95%/2%)	FDA EMA PMDA FDA EMA PMDA	2018-01-12 2019-04-08 2018-01-19 2018-10-16 2019-06-20 2024-01-18
Pancreatic cancer	Olaparib	555 (45%/47%)	FDA EMA PMDA	2019-12-27 2020-07-03 2020-12-25
Prostate cancer	Olaparib Rucaparib Talazoparib	1,460 (0%/94%) 400 (0%/91%) 261 (0%/96%)	FDA EMA PMDA FDA FDA EMA PMDA	2020-05-19 2020-03-11 2020-12-25 2020-05-15 2023-06-20 2023-11-09 2024-01-18

FDA, United States Food and Drug Administration; EMA, European Medicines Agency; PMDA, Japan Pharmaceuticals and Medical Devices Agency.

First approvals for all four PARP drugs were always for BRCA-mutated cancers of women (ovarian and breast cancers). Extended uses for treating cancers involving the two genders (pancreatic cancer) or cancers of men (prostate cancer) were not granted until 3 years later. Beyond the prevalence of the various cancer types, the typology of first approval indications has certainly affected gender reporting frequencies (with only 6% of all indication-annotated ICSR being assigned to male patients), which is in turn likely to influence the type of adverse events associated with these drugs. With potential further extended uses of PARP drugs to cancers affecting both genders, the ratio of post-marketing spontaneous reports between females and males is expected to be more balanced in the future.

### 2.4 Disproportionality parameters

Three disproportionality parameters were used to identify adverse events as SDR for each individual PARP inhibitor: i) the reporting odds ratio (ROR) and its lower and upper limits of the 95% confidence intervals (ROR05 and ROR95), ii) the proportional reporting ratio (PRR) and its lower and upper limits of the 95% confidence intervals (PRR05 and PRR95), and iii) the empirical Bayesian geometric mean (EBGM) and its the lower limit of the 95% confidence interval (EB05) calculated with openEBGM (Canida and Ihrie, 2017). Adverse events were considered SDR if at least five ICSR were associated with the drug and the corresponding value of PRR05 for the drug-event pair exceeded unity (Slattery et al., 2013).

## 3 Results and discussion

### 3.1 SDR for PARP inhibitors

Poly-ADP ribose polymerases (PARP) are a family of enzymes known to play important roles in regulating a wide variety of molecular processes, including DNA repair, gene transcription, cell cycle progression and cell death (Martínez-Bosch et al., 2016; Gupte et al., 2017). Since 2014, four PARP drugs have been approved, namely, olaparib, rucaparib, niraparib, and talazoparib, and at least three more, veliparib, pamiparib, and senaparib have clinical trials ongoing (Makin, 2021). PARP drugs are currently one of the most efficacious targeted therapies to treat ovarian and breast cancers, particularly in women with BRCA1 and/or BRCA2 mutations (Mehta and Bothra, 2021). However, they are not exempt of side effects (LaFargue et al., 2019; Valabrega et al., 2021; Cecere et al., 2023). Among them, fatigue and asthenia are two of the most frequent side effects (55%–65%) encountered during clinical trials (Zhou et al., 2017; DailyMed, 2024). Symptoms of feeling tired, physical weakness and loss of strength (all associated with fatigue and asthenia) could have their origin in haematological events. Indeed, anaemia/hemoglobin decreased (36%–90%), thrombocytopenia/platelet count decreased (10%–60%) and neutropenia/neutrophil count decreased (14%–68%) are also among the most common adverse events detected during the clinical trials of all PARP drugs (Zhou et al., 2017; DailyMed, 2024). Accordingly, these five recognized class-generic clinical adverse drug reactions (ADRs) were selected for further analysis with post-marketing SRS.

In contrast, other adverse events have been so far mostly associated to individual drugs. For example, while insomnia is a common side effect in patients treated with rucaparib (19%) and niraparib (23%), very low or none sleep disorders are found in patients on olaparib and talazoparib (Pagkali et al., 2022; DailyMed, 2024). Olaparib and niraparib have been also associated with pneumonitis (0.8%–2%) and small intestinal obstruction (2.9%–7%), respectively, whereas no association with these potentially serious adverse reactions has been found in clinical trials for the rest of the drugs in the class (Ma et al., 2021; Moore et al., 2019; DailyMed, 2024). Therefore, these three drug-specific clinical ADRs were also added to the selection of events for a focused analysis with post-marketing ICSR databases.

The results of the disproportionality analysis performed on all drug-event pairs are collected in Table 2. As can be observed, all five class-generic clinical ADRs are recovered as post-marketing SDR for all four PARP marketed drugs. The same applies to the investigational drug veliparib, with only asthenia being right below the limits of SDR calling (PRR05 = 0.9). However, fatigue and asthenia are reported more frequently, and more disproportionally compared to the rest of events for each drug, in association with rucaparib and niraparib than with olaparib and talazoparib, whereas the opposite is true for anaemia, thrombocytopenia and neutropenia, which are found generally more frequently, and more disproportionally compared to the rest of events for each drug, in association with olaparib and talazoparib than with rucaparib and niraparib.

**TABLE 2:** Reporting ratios (RR), reporting odds ratios (ROR with 95% two-sided CI), proportional reporting ratios (PRR with 95% two-sided CI) and empirical Bayesian geometric mean (EBGM with the lower limit of the 95% CI) for selected adverse events associated with PARP drugs.

Adverse event	Rucaparib (Rubraca)		Niraparib (Zejula)		Olaparib (Lynparza)		Talazoparib (Talzenna)		Veliparib (ABT-888)	
	RR	ROR (95% CI) PRR (95% CI) EBGM (EB05)	RR	ROR (95% CI) PRR (95% CI) EBGM (EB05)	RR	ROR (95% CI) PRR (95% CI) EBGM (EB05)	RR	ROR (95% CI) PRR (95% CI) EBGM (EB05)	RR	ROR (95% CI) PRR (95% CI) EBGM (EB05)
Fatigue	31.30%	14.1 (13.7–14.5)	22.93%	9.20 (9.01–9.40)	7.97%	2.67 (2.55–2.79)	4.74%	1.53 (1.26–1.86)	7.59%	2.53 (1.98–3.22)
		9.98 (9.67–10.3)		7.32 (7.16–7.48)		2.54 (2.43–2.65)		1.51 (1.24–1.83)		2.41 (1.89–3.07)
		9.98 (9.68)		7.33 (7.18)		2.55 (2.45)		1.50 (1.27)		2.36 (1.90)
Asthenia	8.35%	4.99 (4.66–5.35)	8.52%	5.11 (4.91–5.31)	3.58%	2.03 (1.90–2.18)	4.98%	2.87 (2.39–3.46)	2.53%	***1.42 (0.92–2.19)***
		4.66 (4.35–4.99)		4.76 (4.58–4.95)		2.00 (1.87–2.14)		2.77 (2.30–3.35)		***1.41 (0.91–2.17)***
		4.65 (4.37)		4.75 (4.59)		2.00 (1.89)		2.73 (2.32)		***1.35 (0.92)***
Anaemia	7.42%	8.27 (7.68–8.90)	8.83%	10.0 (9.66–10.4)	15.41%	18.9 (18.4–19.5)	21.63%	28.5 (26.3–30.8)	23.52%	31.7 (28.1–35.7)
		7.73 (7.18–8.32)		9.24 (8.89–9.60)		16.2 (15.7–16.7)		22.5 (20.8–24.4)		24.5 (21.6–27.7)
		7.66 (7.18)		9.17 (8.86)		16.0 (15.6)		21.8 (20.1)		22.4 (19.8)
Thrombocytopenia	2.69%	3.43 (3.02–3.89)	8.23%	11.2 (10.8–11.7)	2.68%	3.42 (3.16–3.70)	11.82%	16.6 (14.8–18.7)	7.21%	9.64 (7.51–12.4)
		3.36 (2.97–3.91)		10.4 (10.0–10.8)		3.35 (3.10–3.63)		14.8 (13.1–16.6)		9.02 (7.02–11.6)
		3.33 (2.99)		10.2 (9.89)		3.33 (3.12)		14.2 (12.8)		8.14 (6.51)
Neutropenia	1.52%	2.22 (1.88–2.63)	1.96%	2.88 (2.65–3.14)	2.12%	3.12 (2.86–3.42)	6.79%	10.5 (8.96–12.3)	5.56%	8.50 (6.38–11.3)
		2.21 (1.86–2.61)		2.85 (2.62–3.10)		3.08 (2.82–3.37)		9.85 (8.41–11.6)		8.08 (6.06–10.8)
		2.18 (1.89)		2.83 (2.64)		3.06 (2.84)		9.40 (8.17)		7.17 (5.56)
Insomnia	3.23%	2.78 (2.48–3.12)	12.61%	12.1 (11.7–12.5)	0.63%	***0.53 (0.45–0.63)***	***0.14%***	-	***0.51%***	-
		2.72 (2.43–3.05)		10.7 (10.4–11.1)		***0.53 (0.45–0.63)***		-		-
		2.70 (2.45)		10.6 (10.33)		***0.53 (0.46)***		-		-
Intestinal obstruction	1.07%	8.60 (7.03–10.5)	1.51%	12.3 (11.2–13.6)	0.55%	4.43 (3.72–5.28)	***0.05%***	-	0.63%	5.04 (2.11–12.0)
		8.52 (6.96–10.4)		12.1 (11.0–13.4)		4.41 (3.70–5.26)		-		5.02 (2.09–12.0)
		8.03 (6.75)		11.9 (10.9)		4.31 (3.71)		-		2.98 (1.36)
Pneumonitis	0.09%	***0.83 (0.42–1.67)***	0.23%	2.15 (1.68–2.75)	0.70%	6.44 (5.50–7.53)	0.43%	3.96 (2.06–7.61)	***0.25%***	-
		***0.83 (0.42–1.67)***		2.14 (1.67–2.74)		6.40 (5.47–7.48)		3.95 (2.06–7.58)		-
		***0.78 (0.42)***		2.09 (1.69)		6.20 (5.43)		3.04 (1.71)		-

Events not fulfilling either of the two conditions for being considered signals of disproportionate reporting (PRR05 > 1.0 and number of reports ≥5) for a particular drug are highlighted in bold italic.

Aligned with clinical findings, relatively high reporting frequencies of insomnia for rucaparib (3.2%) and niraparib (12.6%) are found, which translates in a probability of reporting insomnia rather than any other event approximately over two (ROR05 = 2.5; PRR05 = 2.4; EB05 = 2.5) and ten (ROR05 = 11.7; PRR05 = 10.4; EB05 = 10.3) times higher, respectively, compared to the probability for reference drugs. For niraparib, these values agree well with those reported in an earlier study (Guo et al., 2022).

As regards to intestinal obstruction, even though reporting ratios are below 2% for all drugs, probabilities of reporting the event rather than any other event are high enough (PRR05 > 1.0) to consider it a SDR for all PARP inhibitors, except for talazoparib. This is markedly different than the clinical findings described above in which olaparib was the only PARP drug with small intestinal obstruction being reported during clinical trials. The disproportionality values obtained here for niraparib are aligned with those reported earlier (Guo et al., 2022).

Finally, pneumonitis is rarely reported in association with PARP drugs (all frequencies are well below 1%) but it is found more disproportionally reported, when compared with the rest of events reported for each drug, in association with olaparib and talazoparib than with rucaparib and niraparib.

### 3.2 Source biases in PARP inhibitors

The frequencies of patient sex, reporter types and world regions from the ICSR of each PARP inhibitor, alongside with median vigiGrade completeness scores, are collected in Table 3. Since PARP drugs target mainly cancers of women, there is a general reporting bias towards females. Regarding reporters, different trends in reporter bias are identified across the drug class. While consumers are the most frequent reporters of adverse events for rucaparib and niraparib, physicians are the main reporters for olaparib, talazoparib, and veliparib. Of note is the presence of 24 reports from consumers of veliparib, likely participants in some of the many clinical trials of this investigational drug since 2009. Finally, while most reports for rucaparib, niraparib, and veliparib come from the American region, no reporting bias between the American and the European regions is observed for olaparib and talazoparib. In summary, PARP drugs can be split in two groups based on the reporting patterns observed: rucaparib and niraparib, on one side, and olaparib and talazoparib, on another side. Veliparib sits currently between the two with a mixed source reporting pattern.

**TABLE 3:** Patient sex, case reporter and case region frequencies from individual case safety reports (ICSR) of each of the five PARP inhibitors. Median vigiGrade scores are included to reflect the overall content completeness of all ICSR for each drug.

Characteristic	Rucaparib (Rubraca)	Niraparib (Zejula)	Olaparib (Lynparza)	Talazoparib (Talzenna)	Veliparib (ABT-888)
ICSR	8,806	26,954	22,432	2.090	791
Median vigiGrade	0.39	0.26	0.35	0.46	0.51
Patient Sex					
Female	7,775 (88.3%)	18,724 (69.5%)	17,565 (78.3%)	1,512 (72.3%)	445 (56.3%)
Male	537 (6.1%)	409 (1.5%)	3,116 (13.9%)	409 (19.6%)	91 (11.5%)
Not reported	494 (5.6%)	7,821 (29.0%)	1,751 (7.8%)	169 (8.1%)	255 (32.2%)
Case reporter					
Consumer	3,029 (34.4%)	14,769 (54.6%)	5,878 (26.2%)	373 (17.8%)	24 (3.0%)
Physician	1,129 (12.8%)	8,738 (32.3%)	10,969 (48.9%)	1,256 (60.1%)	550 (69.5%)
Pharmacist	159 (1.8%)	884 (3.3%)	1,075 (4.8%)	69 (3.3%)	6 (0.8%)
Other health professionals	3,012 (34.2%)	527 (1.9%)	408 (1.8%)	39 (1.9%)	135 (17.1%)
Lawyer	0 (0.0%)	4 (0.0%)	3 (0.0%)	0 (0.0%)	0 (0.0%)
Not reported	1,477 (16.8%)	2,151 (7.9%)	4,114 (18.3%)	353 (16.9%)	76 (9.6%)
Case Region					
American	7,780 (88.3%)	17,989 (66.8%)	8,175 (36.5%)	903 (43.2%)	579 (73.2%)
European	940 (10.7%)	5,267 (19.5%)	7,758 (34.6%)	1,020 (48.8%)	155 (19.6%)
Western Pacific	77 (0.9%)	3,689 (13.7%)	4,708 (21.0%)	109 (5.2%)	48 (6.1%)
African	0 (0.0%)	0 (0.0%)	29 (0.1%)	4 (0.2%)	8 (1.0%)
Eastern Mediterranean	0 (0.0%)	4 (0.0%)	203 (0.9%)	23 (1.1%)	1 (0.1%)
South-East Asia	7 (0.1%)	1 (0.0%)	1,530 (6.8%)	29 (1.4%)	0 (0.0%)
Not reported	2 (0.0%)	4 (0.0%)	29 (0.1%)	2 (0.1%)	0 (0.0%)

### 3.3 Source biases in SDR for PARP inhibitors

Along the same lines, the frequencies of patient sex, reporter types and world regions from the ICSR of the eight selected SDR are collected in Tables 4, 5. Interestingly, different source patterns are also observed. Fatigue, asthenia, insomnia, and intestinal obstruction are generally more reported by consumers than by physicians and also those reports originate more frequently in America than in Europe. In marked contrast, physicians are the main reporters of anaemia, thrombocytopenia, neutropenia, and pneumonitis and, in those cases, reports generally originate more frequently in Europe than in America. Accordingly, SDR can be split in two groups based on the reporting patterns observed: fatigue, asthenia, insomnia, and intestinal obstruction, on one side, and anaemia, thrombocytopenia, neutropenia and pneumonitis, on another side.

**TABLE 4:** Patient sex, reporter type and world region frequencies from individual case safety reports (ICSR) of fatigue, insomnia, asthenia and intestinal obstruction. Median vigiGrade scores are included to reflect the overall content completeness of all ICSR for each event.

Characteristic	Fatigue	Asthenia	Insomnia	Intestinal obstruction
ICSR	1,982,167	943,432	521,934	49,991
Median vigiGrade	0.46	0.43	0.36	0.36
Patient Sex				
Female	1,187,366 (59.9%)	465,013 (49.3%)	246,858 (47.3%)	19,113 (38.2%)
Male	528,584 (26.7%)	267,353 (28.3%)	134,285 (25.7%)	15,628 (31.3%)
Not reported	266,217 (13.4%)	211,066 (22.4%)	140,791 (27.0%)	15,250 (30.5%)
Case Reporter				
Consumer	985,868 (49.7%)	339,264 (35.9%)	251,359 (48.1%)	18,276 (36.5%)
Physician	265,691 (13.4%)	216,414 (22.9%)	88,825 (17.0%)	13,815 (27.5%)
Pharmacist	89,247 (4.5%)	50,977 (5.4%)	32,456 (6.2%)	2,275 (4.5%)
Other professionals	73,045 (3.7%)	46,020 (4.9%)	23,202 (4.5%)	4,438 (8.9%)
Lawyer	10,169 (0.5%)	7,444 (0.8%)	5,904 (1.1%)	2,057 (4.1%)
Not reported	558,336 (28.2%)	283,694 (30.1%)	120,355 (23.1%)	9,296 (18.5%)
Case Region				
American	885,037 (44.6%)	460,158 (48.8%)	304,000 (58.3%)	32,758 (65.5%)
European	800,392 (40.4%)	280,121 (29.7%)	128,607 (24.6%)	8,888 (17.8%)
Western Pacific	78,555 (4.0%)	100,693 (10.7%)	52,159 (10.0%)	6,414 (12.8%)
African	18,956 (1.0%)	16,199 (1.7%)	4,846 (0.9%)	173 (0.4%)
Eastern Mediterranean	75,785 (3.8%)	17,958 (1.9%)	5,878 (1.1%)	263 (0.5%)
South-East Asia	14,129 (0.7%)	18,298 (1.9%)	7,619 (1.5%)	271 (0.5%)
Not reported	109,313 (5.5%)	49,735 (5.3%)	18,825 (3.6%)	1,224 (2.5%)

**TABLE 5:** Patient sex, reporter type and world region frequencies from individual case safety reports (ICSR) of anaemia, pneumonitis, thrombocytopenia and neutropenia. Median vigiGrade scores are included to reflect the overall content completeness of all ICSR for each event.

Characteristic	Anaemia	Thrombocytopenia	Neutropenia	Pneumonitis
ICSR	381,484	325,911	272,112	43,951
Median vigiGrade	0.41	0.39	0.45	0.40
Patient Sex				
Female	151,351 (39.7%)	113,623 (34.9%)	117,627 (43.2%)	16,175 (36.8%)
Male	113,955 (29.9%)	120,453 (36.9%)	86,984 (32.0%)	15,939 (36.3%)
Not reported	116,178 (30.4%)	91,835 (28.2%)	67,501 (24.8%)	11,837 (26.9%)
Case Reporter				
Consumer	70,931 (18.5%)	30,867 (9.4%)	18,317 (6.7%)	5,940 (13.5%)
Physician	174,389 (45.5%)	145,119 (44.4%)	146,257 (53.5%)	22,392 (50.7%)
Pharmacist	31,421 (8.2%)	27,637 (8.5%)	29,040 (10.6%)	2,578 (5.8%)
Other professionals	35,226 (9.2%)	26,820 (8.2%)	25,539 (9.4%)	4,284 (9.7%)
Lawyer	4,015 (1.1%)	802 (0.3%)	255 (0.1%)	173 (0.4%)
Not reported	66,903 (17.5%)	95,445 (29.2%)	53,876 (19.7%)	8,774 (19.9%)
Case Region				
American	145,688 (38.2%)	84,629 (26.0%)	69,537 (25.6%)	15,583 (35.4%)
European	135,408 (35.5%)	139,953 (42.9%)	121,775 (44.8%)	17,348 (39.5%)
Western Pacific	64,540 (16.9%)	80,643 (24.7%)	67,596 (24.8%)	8,648 (19.7%)
African	4,897 (1.3%)	599 (0.2%)	1,019 (0.4%)	123 (0.3%)
Eastern Mediterranean	5,021 (1.3%)	3,849 (1.2%)	3,087 (1.1%)	255 (0.6%)
South-East Asia	16,299 (4.3%)	7,064 (2.2%)	4,530 (1.6%)	428 (1.0%)
Not reported	9,631 (2.5%)	9,174 (2.8%)	4,568 (1.7%)	1,566 (3.5%)

The results agree well with previous studies on the reporting differences between consumers and physicians (Aagaard et al., 2009; Al Dweik et al., 2020). In those works, odds ratios (OR) were used to show that consumers were indeed found to report more than other health professionals adverse events from general disorders and administration site conditions (OR values of 1.10 in both works), such as fatigue and asthenia, and psychiatric disorders (OR values of 1.70 and 2.20), such as insomnia. In contrast, consumers tend to report less than other sources adverse events from blood and lymphatic system disorders (OR values of 0.22 and 0.21), such as anaemia, thrombocytopenia and neutropenia, and respiratory, thoracic, and mediastinal disorders (OR values of 0.81 and 0.86), such as pneumonitis. For gastrointestinal disorders, such as intestinal obstruction, conflicting trends between different reporting sources were found (OR values of 1.24 and 0.88).

### 3.4 Source biases in individual ICSR databases

A comparative analysis of the unique ICSR from FAERS and VigiBase, the two largest SRS used in this work, reveals that while FAERS contains more than double the number of reports from consumers (46.3%) than from physicians (21.4%) and five times more reports from America (76.4%) than from Europe (14.6%), the opposite is true for VigiBase, with reports from physicians (33.6%) more than doubling reports from consumers (16.3%) and reports from Europe (42.1%) being almost two and a half times those from America (17.9%). Comparatively, consumer reports from FAERS are more than two and a half times those from VigiBase and physician reports from VigiBase almost double those from FAERS, whereas American reports from FAERS are almost four times more than those from VigiBase and European reports from VigiBase exceed almost three times those from FAERS. Therefore, FAERS and VigiBase appear to be biased with reports from American consumers and European physicians, respectively, and they are thus complimentary in this respect. With respect to reporter types, JADER is an extreme case as it contains almost ten times more reports from physicians (78.4%) than from consumers (8.1%).

Since it has been shown that consumers and physicians report adverse events differently (Aagaard et al., 2009; Al Dweik et al., 2020), variances in reporting patterns from individual ICSR databases may affect the results of disproportionality analyses and ultimately the detection of SDR for a given drug. For example, we found that niraparib reports come mainly from American consumers (Table 3). A similar pattern is recovered using either FAERS or VigiBase individually, although FAERS naturally contains more niraparib reports than VigiBase from consumers (63.3% vs*.* 56.1%) and from the American region (80.9% vs*.* 71.1%). Similar disproportionality measures are thus obtained with both FAERS and VigiBase. In contrast, of a total of 1,635 reports for niraparib found in JADER, 1.1% are deposited by consumers and 87.7% by physicians. Such an opposite reporting pattern compared with the other two ICSR databases may have influenced the lower disproportionality values obtained for adverse events that usually tend to be reported more frequently by consumers than by physicians. This is the case of insomnia, for which only 1 report is available in JADER and thus it will not be a SDR for niraparib (PRR05 = 10.4 in Table 2), and intestinal obstruction, with PRR05 = 1.89 (compared with PRR05 = 11.0 in Table 2).

Same trends are found for olaparib. In this case, the results from the integrated ICSR database indicated that adverse events for olaparib are more frequently reported by physicians than by consumers (Table 3). Again, the same trend is recovered using either FAERS or VigiBase individually, although in this case VigiBase naturally contains more olaparib reports than FAERS from physicians (42.8% vs*.* 39.7%) and from the European region (38.1% vs*.* 20.0%). Despite these differences in relative reporting frequencies, disproportionality measures obtained with both FAERS and VigiBase were comparable. However, of the total numberof 1,691 olaparib reports found in JADER, 0.8% are from consumers and 93.7% from physicians. The reporting gap between consumers and physicians in JADER is much wider than the one found with the other two ICSR databases and thus, an impact on the disproportionality values obtained for adverse events usually more frequently reported by consumers than by physicians should be expected. This is the case of asthenia, for which only 1 report is present in JADER and thus it will not be a SDR for olaparib (PRR05 = 1.87 in Table 2), and intestinal obstruction, with PRR05 = 1.59 (compared with PRR05 = 3.70 in Table 2).

### 3.5 Differential source pattern analysis

**FIGURE 1:**
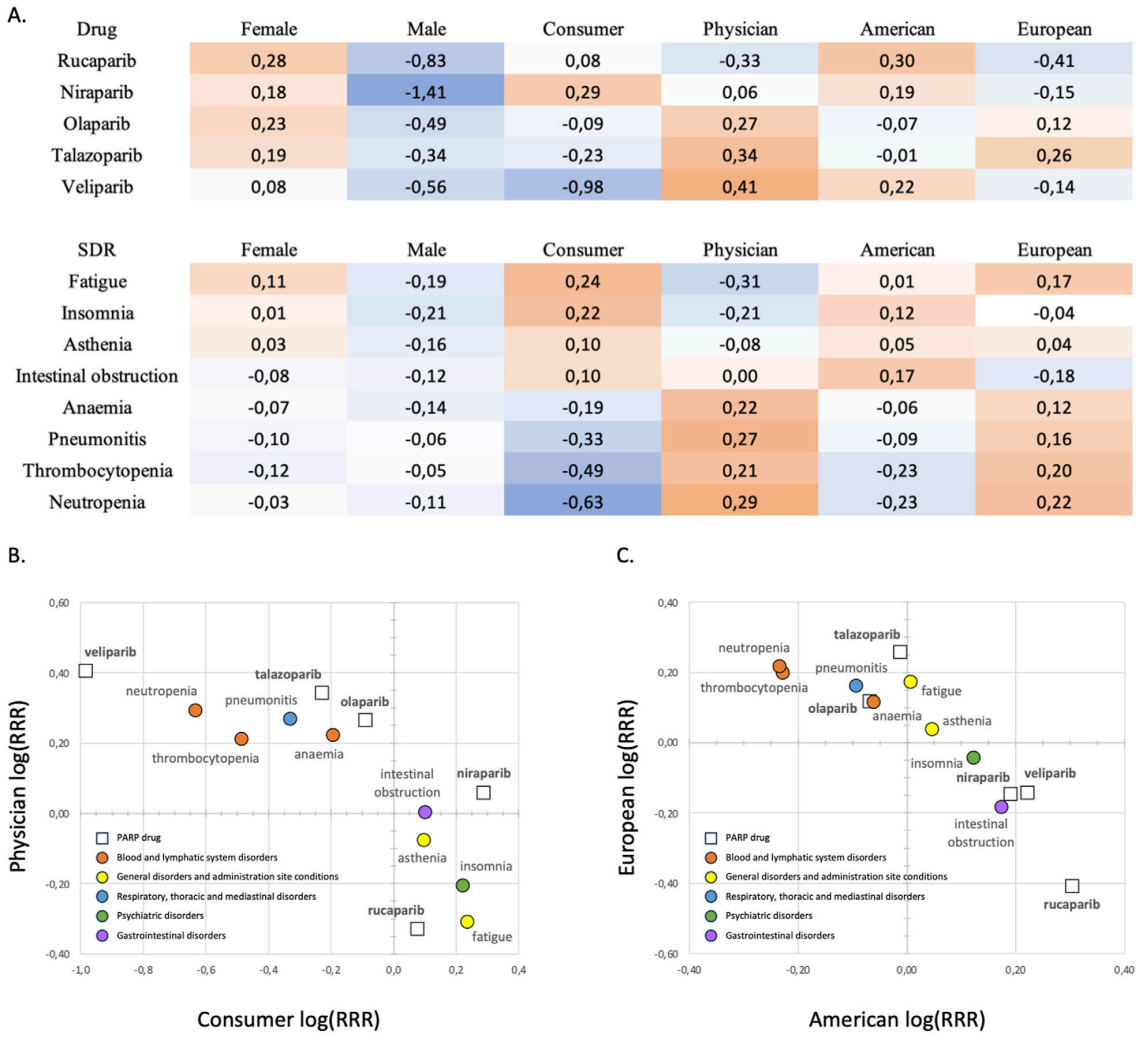
**(A)** Logarithmic values of relative reporting ratios, log (RRR), **(B)** differential reporter patterns and **(C)** differential region patterns for the five PARP inhibitors and the selection of eight adverse events. Red and blue cells indicate positive and negative log (RRR) values, respectively, relative to the background reporting frequencies.

Matched reporting patterns between drugs and events may predispose certain drugs to be disproportionally associated with adverse events. To investigate the influence of differential source patterns in the detection of SDR for PARP inhibitors, reporting frequencies of patient sex (females and males), reporter type (consumers and physicians) and geographic region (American and European) obtained for the five PARP inhibitors (Table 3) and the selection of eight adverse events (Tables 4, 5) where compared with background reporting frequencies obtained from the total number of 55,751,007 unique ICSR. The results are illustrated in Figure 1 in which, for the ease of interpretation, relative reporting ratios (RRR) are expressed in logarithmic values, log (RRR).

As can be observed in Figure 1A, relative reporting ratios for females and males obtained for all five PARP inhibitors are respectively higher, log (RRR) > 0, and lower, log (RRR) < 0, than the corresponding background frequencies (46.4% for females and 41.3% for males). This is an expected result considering that PARP drugs are mainly used as first-line treatments in ovarian and breast cancers. This class-wide sex reporting pattern matches well with the pattern observed for fatigue, asthenia and insomnia. However, for the other five adverse events, both females and males appear under-reported, an outcome that reflects the presence of a relatively high proportion of reports without information on patient sex for those events (13.4%–30.5%; see Tables 4, 5).

The same analysis for consumers and physicians differentiates drugs and events in two main groups: on one side, the drugs rucaparib and niraparib, and the adverse events fatigue, asthenia, insomnia and intestinal obstruction, show higher reporting ratios for consumers relative to physicians than the corresponding background frequencies (28.8% and 27.3% for consumers and physicians, respectively); on the other side, the drugs olaparib, talazoparib and veliparib, and the adverse events anaemia, thrombocytopenia, neutropenia and pneumonitis, have higher relative reporting ratios for physicians than consumers.

The results of relative reporting ratios for the American and European regions return also two groups: the drugs rucaparib, niraparib and veliparib, and the adverse events insomnia and intestinal obstruction, show higher reporting ratios for the American region relative to the European region than the corresponding background frequencies (43.9% and 27.2% for the American and European regions, respectively); on the other side, the drugs olaparib and talazoparib, and the adverse events anaemia, thrombocytopenia, neutropenia and pneumonitis, have higher relative reporting ratios for the European region than the American region. Fatigue and asthenia show slightly increased relative reporting ratios compared to the background for both the American and European regions.

To gain a more visual perception of those drug-event groups, the values displayed in Figure 1A are plotted in Figures 1B, C. The plot in the reporter space (Figure 1B) illustrates clearly that rucaparib and niraparib have similar reporting patters to fatigue, asthenia, insomnia, and intestinal obstruction, whereas olaparib and talazoparib are placed closer to the reporting patterns of anaemia, thrombocytopenia, neutropenia and pneumonia. In turn, the corresponding plot in the region space (Figure 1C) places niraparib and veliparib in the vicinity of insomnia and intestinal obstruction, whereas olaparib and talazoparib are close to the three haematological events and pneumonitis, with fatigue and asthenia sitting between the two groups.

Since niraparib is more frequently reported by consumers (54.6%) than by physicians (32.3%) and those reports originate more frequently from the region of America (66.8%) than in Europe (19.5%), this drug is in a good position, compared to other drugs with reversed reporting patterns, to achieve disproportional reporting of events with similar reporting patterns, such as insomnia, also more frequently reported by consumers (48.1%) than by physicians (17.0%) and from the region of America (58.3%) than in Europe (24.6%). Therefore, the fact that rucaparib and niraparib are most frequently reported by American consumers, and American consumers are the main reporters of insomnia, could partly explain the rapid detection of insomnia as a post-marketing SDR for these two drugs but not for the other three PARP inhibitors (Table 2). Similarly, the fact that olaparib and talazoparib are most frequently reported by European physicians, and European physicians are the main reporters of pneumonitis could partly explain the rapid detection of pneumonitis as a post-marketing SDR for these two drugs compared to the rest (Table 2). However, a more detailed analysis of the reporting patterns from different sources is required to identify the origin of the disproportional reporting.

Table 6 presents a source subgroup disproportionality analysis of the ICSR of five adverse events (one representative of each SOC). Taking the case highlighted above of niraparib and insomnia, 83.0% of niraparib reports for insomnia were reported by consumers against 9.0% by physicians, and 93.8% originated in America in contrast to only 5.2% in Europe. The reporting frequencies from consumers and America are both clearly higher than the corresponding background frequencies for insomnia (Table 4), resulting in disproportionality measures above one (ROR values of 1.73 and 1.62, respectively). As a reference, the reporting frequencies from consumers and America for niraparib are 54.6% and 66.8%, respectively (Table 2), both values already above the background frequencies for insomnia (48.1% for consumers and 58.3% for the American region). The result is that insomnia is a SDR for niraparib (ROR = 12.1; see Table 2).

**TABLE 6:** Reporting ratios (RR) and reporting odd ratios (ROR) of selected reporters (consumer and physician) and regions (American and European) for five selected adverse events associated with PARP inhibitors. Subgroups with less than 5 reports are marked with a “-”.

Comparator Subgroup	Rucaparib (Rubraca)		Niraparib (Zejula)		Olaparib (Lynparza)		Talazoparib (Talzenna)		Veliparib (ABT-888)	
	RR	ROR	RR	ROR	RR	ROR	RR	ROR	RR	ROR
Fatigue										
Consumer	34.58%^1^	0.69	82.98%	1.67	33.72%	0.68	23.23%	0.47	—	—
Physician	11.68%^1^	0.87	8.69%	0.65	39.43%	2.95	48.48%	3.62	78.33%	5.84
American	96.88%	2.17	93.92%	2.11	48.77%	1.09	50.51%	1.13	75.00%	1.68
European	2.94%	0.07	5.44%	0.13	37.25%	0.92	45.45%	1.13	20.00%	0.50
Insomnia										
Consumer	37.68%^1^	0.78	83.02%	1.73	50.70%	1.05	—	—	—	—
Physician	10.56%^1^	0.67	9.03%	0.53	19.72%	1.16	—	—	—	—
American	98.59%	1.69	93.76%	1.62	60.56%	1.04	—	—	—	—
European	1.41%	0.06	5.24%	0.21	18.31%	0.74	—	—	—	—
Intestinal obstruction										
Consumer	27.66%^1^	0.76	68.38%	1.88	34.68%	0.95	—	—	—	—
Physician	13.83%^1^	0.50	20.59%	0.74	40.32%	1.46	—	—	—	—
American	77.66%	1.19	84.56%	1.29	37.10%^3^	0.57	—	—	—	—
European	20.21%	1.14	6.62%	0.37	12.90%^3^	0.73	—	—	—	—
Thrombocytopenia										
Consumer	22.36%^1^	2.36	25b17%	2.69	7.65%	0.81	3.64%	0.38	—	—
Physician	28.27%^1^	0.63	59.27%	1.33	68.39%	1.54	82.19%	1.85	57.89%^5^	1.30
American	72.57%	2.80	34.91%	1.35	22.63%	0.87	13.36%	0.51	77.19%	2.97
European	25.d2%	0.59	54.08%	1.26	66.39%	1.55	79.35%	1.85	14.04%	0.33
Pneumonitis										
Consumer	—	—	31.75%	2.35	7.69%	0.57	—	—	—	—
Physician	—	—	58.73%	1.15	67.95%	1.34	77.78%	1.53	—	—
American	62.50%	1.76	47.62%	1.34	44.23%	1.25	—^4^	—	—	—
European	—^2^	—	44.44%	1.13	39.74%	1.01	55.56%	1.41	—	—

1 Other health professionals accounted for 38.79% (ROR, 10.48), 36.97% (ROR, 8.22), 39.36% (ROR, 4.42), and 31.65% (ROR, 3.86) of the reports for fatigue, insomnia, intestinal obstruction, and thrombocytopenia, respectively.

2 The European region accounted for 3 of the 8 reports (37.50%) for pneumonitis.

3 The Western Pacific region accounted for 37.90% (ROR, 2.96) of the reports for intestinal obstruction.

4 The American region accounted for 3 of the 9 reports (33.33%) for pneumonitis.

5 Other health professionals accounted for 26.32% (ROR, 3.21) of the reports for thrombocytopenia.

A different scenario is the one faced by olaparib. In this case, olaparib has basal reporting frequencies for consumers and America of 26.2% and 36.5% (Table 3), both values much lower than the corresponding basal frequencies of 48.1% and 58.3% for insomnia (Table 4). Even though the consumer and America reporting of insomnia for olaparib increased to 50.7% and 60.6% (ROR values of 1.05 and 1.04, respectively), reversing the original reporting trend for the drug and approaching the source pattern for the event was not sufficient for insomnia to become a SDR for olaparib (ROR = 0.53; see Table 2), even though the reporting of insomnia from physicians was also above the expected ratio (ROR = 1.16).

In contrast, we see that 68.0% of olaparib reports for pneumonitis were reported by physicians against 7.7% by consumers, and 44.2% originated in America compared to 39.7% in Europe. The reporting frequency by physicians (68.0%) is higher than the corresponding background frequency for pneumonitis (50.7%; see Table 5), resulting in a disproportionality measure above one (ROR = 1.34). As a reference, the reporting frequency by physicians for olaparib is 48.9% (Table 2), a value already very close to the background frequency for pneumonitis (50.7%; see Table 5). Interestingly, reporting of pneumonitis for olaparib from the American region is also higher than what would be expected for this event (ROR = 1.25). The result is that pneumonitis is a SDR for olaparib (ROR = 6.44; see Table 2).

One may argue that the most interesting cases emerge when drugs and events have opposed source patterns and, nonetheless, in the ICSR of the drug for the event the drug reporting pattern is reversed to the extent that the event becomes a SDR. For example, the niraparib basal reporting frequency for physicians is 32.3% (Table 3), a value well below the corresponding background frequency of 50.7% for pneumonitis (Table 5). Interestingly, the basal reporting pattern of niraparib was reversed when reporting pneumonitis and the percentage of physicians reporting pneumonitis increased to 58.7% (ROR = 1.15) compared with a consumer reporting of 31.8%, much higher than the basal reporting frequency for pneumonitis (13.5%; ROR = 2.35). In addition, the basal region bias for niraparib was also reversed and the European reporting of pneumonitis increased from 19.5% (Table 3) to 44.4%, already above the background European reporting for that event (39.5%; ROR = 1.13). Therefore, despite their opposed basal reporting patterns, the disproportional reporting of niraparib ICSR for pneumonitis from all sources (both reporters and regions) ultimately resulted in pneumonitis being a SDR for niraparib (ROR = 2.15; see Table 2).

Reversed reporting patterns are generally observed across all drugs for class events, such as fatigue and thrombocytopenia. For example, olaparib and talazoparib are mainly reported by physicians (Table 3) and fatigue by consumers (Table 4) yet fatigue is detected as a SDR in both drugs (Table 2). As can be observed in Table 6, the physician reporting of fatigue for olaparib is 39.4%, 9.5% below the basal physician reporting frequency for the drug (Table 3) but 26.0% above the expected physician reporting for the event (ROR = 2.95). The over-reporting of the event by physicians overcomes the under-reporting of consumers (33.7%) that, even though it is 7.5% above the basal consumer reporting frequency for olaparib (Table 3) it still is 16.0% below the expected consumer reporting for the event (ROR = 0.68). The overall result is that fatigue is detected as a SDR for olaparib (ROR = 2.67; see Table 2). Along the same lines but in the other direction, rucaparib and niraparib are mainly reported by American consumers (Table 3) and thrombocytopenia by European physicians (Table 5) yet thrombocytopenia is clearly detected as a SDR in both drugs (Table 2). As can be observed in Table 6, the consumer reporting of thrombocytopenia for niraparib is 25.2%, 29.4% below the basal consumer reporting frequency for the drug (Table 3) but 15.8% above the expected consumer reporting for the event (ROR = 2.69), and at the same time the physician reporting is 59.3%, 27.0% above the basal physician reporting frequency for niraparib (Table 3) and 14.9% also above the expected physician reporting for the event (ROR = 1.33). Altogether results in thrombocytopenia being detected as a SDR for niraparib (ROR = 11.2; see Table 2).

### 3.6 Influence of report completeness on SDR

One final aspect that deserves some consideration is the robustness of SDR detection upon data completeness. Despite being often overlooked, it is relevant in this case because we observed relatively high levels of missing ICSR data in all characteristics being analysed across all drugs and events. Among the five PARP inhibitors (Table 3), levels of missing data varied widely for patient sex, between 5.6% (in rucaparib) and 32.2% (in veliparib), and case reporter, between 7.9% (in niraparib) and 18.3% (in olaparib), whereas data for case region showed remarkably high levels of completeness (above 99.8%). Similar trends were found among the list of eight selected adverse events (Tables 4, 5) where average levels of empty fields for patient sex, case reporter and case region were 25.5%, 23.3%, and 3.4%, respectively. Given these results, the potential impact of missing data on SDR detection for PARP drugs deserves some consideration.

VigiGrade scores were used to quantify the level of data completeness in ICSR for drugs and events. It was already shown above (Table 3) that median vigiGrade values across the ICSR of PARP inhibitors ranged from 0.26 in niraparib, the most consumer-biased drug, to 0.51 in veliparib, the most physician-biased drug. In agreement in previous observations (Bergvall et al., 2014), this indicates that consumer reports are generally less complete than reports deposited by physicians. Indeed, the average vigiGrade value of consumer reports among PARP drugs is 0.35 compared to a score of 0.43 found in physician reports. In addition, significant variations in vigiGrade scores were also observed depending on the geographical origins of reports associated with PARP inhibitors. In this respect, average vigiGrade values for the American, European, and Western Pacific regions were 0.34, 0.42, and 0.54, respectively. This is consistent with previous analyses indicating that reports from Europe seem to be better documented than those deposited from the American region (Bergvall et al., 2014) and highlights the superior quality of reports from the Western Pacific region. Similar trends in average vigiGrade completeness scores were found for ICSR of adverse events.

To analyze the influence of report completeness on SDR detection, proportional reporting ratios (PRR) were obtained for subsets of ICSR having vigiGrade values above certain thresholds. Since reports from physician reporters and the European region tend to have higher vigiGrade scores than reports from consumer reports and the American region it is expected that, as we increase the vigiGrade threshold, the subset of remaining ICSR will be enriched with reports deposited by physicians and from the European region. Consequently, different trends on reporting disproportionalities may be observed for adverse events having essentially opposite reporting patterns (Tables 4, 5).

For illustrative purposes, we focused on the results for niraparib and olaparib as representatives of the main reporting patterns observed among PARP drugs (Table 3). As expected, with higher vigiGrade thresholds the relative reporting frequencies of the various case reporters and regions varied following the trends in data completeness highlighted above. For niraparib, taking only ICSR with minimum vigiGrade score of 0.30, the original consumer/physician ratio (54.6%/32.3% in Table 3) was reversed (37.4%/47.8%), and the original relative reporting frequencies for the American/European/WPacific regions (66.8%/19.5%/13.7% in Table 3) were modulated accordingly (47.8%/26.1%/26.1%), due to the enrichment of physician over consumer reports, and those from Western Pacific and European regions over American regions, at higher vigiGrade scores. For olaparib, the effect of using a vigiGrade threshold of 0.30 resulted in a widening of the reporting frequency gap between consumer and physician reports (the original 26.2%/48.9% frequencies in Table 3 become 20.4%/57.1%) and an inversion of the relative frequencies of reports from the American/European/WPacific regions (the original 36.5%/34.6%/21.0% frequencies in Table 3 become 25.9%/36.1%/30.4%).

Having established the alteration of the original reporting patterns when limiting the analysis to a subset of well-documented reports, what is left is to assess the impact on the disproportionality measures calculated for the various drug-event pairs and ultimately on SDR detection. Table 7 collects the proportional reporting ratios (PRR), with two-sided confidence intervals (CI), for the selection of eight adverse events associated with niraparib and olaparib when considering the subset of ICSR with vigiGrade thresholds of 0.00 (all ICSR), 0.15, and 0.30. It is interesting to observe that for niraparib, with a median vigiGrade score of all its ICSR of 0.26 (Table 3), the lowest among all five PARP inhibitors, the change in the reporting pattern as we move towards better documented reports results, on the one hand, in reduced PRR values for adverse events with a consumer-/American-biased reporting pattern (fatigue, insomnia, asthenia, and intestinal obstruction) and, on the other hand, in increased PRR values for those adverse events with physician-/European-biased reporting pattern (anaemia, pneumonitis, thrombocytopenia, neutropenia). In contrast, the effect on olaparib, with a median vigiGrade score of all its ICSR of 0.35 (Table 3) and thus, above the highest vigiGrade threshold considered (0.30), is not so remarkable and PRR values remain reasonably stable across the different vigiGrade thresholds. In any case, at least for the cases studied here, SDR detection seems quite robust to data completeness and none of the adverse events considered lost its SDR status for any of the PARP inhibitors.

**TABLE 7:** Proportional reporting ratios (PRR with 95% two-sided CI) for selected adverse events associated with niraparib and olaparib when considering individual case safety reports (ICSR) above different vigiGrade thresholds. Events not fulfilling either of the two conditions for being considered signals of disproportionate reporting (PRR05 > 1.0 and number of reports ≥5) for a particular drug are highlighted in bold italic.

Adverse event	Niraparib (zejula)			Olaparib (lynparza)		
	vigiGrade ≥0.00	vigiGrade ≥0.15	vigiGrade ≥0.30	vigiGrade ≥0.00	vigiGrade ≥0.15	vigiGrade ≥0.30
	ICSR = 26,954	ICSR = 21,212	ICSR = 10,196	ICSR = 22,432	ICSR = 20.075	ICSR = 11,929
Fatigue	7.32 (7.16–7.48)	6.30 (6.13–6.48)	5.35 (5.13–5.59)	2.54 (2.43–2.65)	2.53 (2.41–2.65)	2.57 (2.42–2.73)
Insomnia	10.07 (10.4–11.1)	9.24 (8.89–9.61)	8.06 (7.59–8.57)	***0.53 (0.45–0.63)***	***0.53 (0.45–0.63)***	***0.58 (0.47–0.72)***
Asthenia	4.76 (4.58–4.95)	4.14 (3.95–4.34)	3.69 (3.44–3.95)	2.03 (1.90–2.18)	2.12 (1.98–2.27)	2.16 (1.99–2.35)
Intestinal obstruction	12.1 (11.0–13.4)	12.2 (11.0–13.6)	11.1 (9.47–13.0)	4.41 (3.70–5.26)	4.52 (3.77–5.40)	4.86 (3.89–6.07)
Anaemia	9.24 (8.89–9.60)	9.34 (8.96–9.74)	10.5 (9.91–11.0)	16.2 (15.7–16.7)	16.8 (16.3–17.33)	17.9 (17.3–18.6)
Pneumonitis	2.14 (1.67–2.74)	2.29 (1.76–2.97)	3.14 (2.29–4.29)	6.40 (5.47–7.48)	6.36 (5.41–7.47)	7.10 (5.85–8.60)
Thrombocytopenia	10.4 (10.0–10.8)	10.9 (10.4–11.3)	12.6 (11.9–13.3)	3.35 (3.10–3.63)	3.41 (3.15–3.69)	3.13 (2.83–3.46)
Neutropenia	2.85 (2.62–3.10)	3.13 (2.87–3.42)	3.60 (3.22–4.03)	3.08 (2.82–3.37)	3.07 (2.80–3.37)	2.86 (2.54–3.22)

## 4 Conclusion

A reporting bias analysis of post-marketing databases revealed that, while some adverse events, such as asthenia and insomnia, are mostly reported by American consumers, other side effects, such as thrombocytopenia and pneumonitis, are mainly reported by European physicians. The fact that rucaparib and niraparib receive most reports from American consumers could disproportionally enhance the reporting rate of events with matching reporting patterns, such as asthenia and insomnia, over other events with opposite reporting patterns. The same would apply for olaparib and talazoparib having matched reporting patterns with events like thrombocytopenia and pneumonitis. Therefore, reporting biases in drugs highlight limitations in the interpretation of disproportionality results for events with matched drug-event reporting patterns, as it may predispose drugs to be disproportionally associated with adverse events. Accordingly, SDR detected from matched drug-event reporting patterns in ICSR databases should be challenged during signal validation.

In addition, the identification of differential reporting patterns between drugs in a class also highlights limitations in interpreting disproportionality results for the same event across drugs within the class. In the case of opposite drug-event patterns, drug source reporting needs to be reversed to approach the event reporting pattern. Class SDR for drugs with differential patterns (such as fatigue, asthenia, anaemia, thrombocytopenia, and neutropenia for PARP inhibitors) usually involve correcting opposite drug-event source patterns. In this respect, it is worth emphasizing that, despite affecting disproportionality measures, SDR detection was found to be robust when challenged against ICSR data completeness.

Overall, it has been shown that the presence of different reporting biases from reporter types and world regions among drugs in a class influences SDR detection. Routine differential source pattern analysis should be part of signal validation when evaluating SDR from ICSR databases.

## Data Availability

Not all datasets used in this article are readily available. The FAERS data that support the findings of this study are publicly available from the FDA website (FAERS 2024). The JADER and VigiBase data used are available from INTAGE Healthcare (INTAGE Healthcare 2024) and WHO-UMC (WHO-UMC 2024), respectively, but restrictions apply to the availability of these data, which were used under license for the current study, and so are not publicly available. For VigiBase, the UMC provided the data, but the study results and conclusions are those of the author and not necessarily those of the UMC, National Centres, or WHO. Requests to access the datasets should be directed to FAERS Quarterly Data Extract Files (2024). https://fis.fda.gov/extensions/FPD-QDE-FAERS/FPD-QDE-FAERS.html (last accessed on August 30th, 2024). VAERS (2024). https://vaers.hhs.gov/data.html (last accessed on August 30th, 2024). JADER (2024). https://www.intage-healthcare.co.jp/english/ (last accessed on August 30th, 2024). VigiBase (2024). https://who-umc.org/vigibase/ (last accessed on August 30th, 2024).
